# Alkaline phosphatase downregulation promotes lung adenocarcinoma metastasis via the c-Myc/RhoA axis

**DOI:** 10.1186/s12935-021-01919-7

**Published:** 2021-04-15

**Authors:** Zhefeng Lou, Weiwei Lin, Huirong Zhao, Xueli Jiao, Cong Wang, He Zhao, Lu Liu, Yu Liu, Qipeng Xie, Xing Huang, Haishan Huang, Lingling Zhao

**Affiliations:** 1 Zhejiang Provincial Key Laboratory of Medical Genetics, Key Laboratory of Laboratory Medicine, Ministry of Education, School of Laboratory Medicine and Life Sciences, Wenzhou Medical University, Wenzhou, 325035 Zhejiang China; 2 The First Affiliated Hospital of Wenzhou Medical University, Wenzhou, 325035 Zhejiang China; 3 Zhejiang Provincial Key Laboratory of Pancreatic Disease, School of Medicine, the First Affiliated Hospital, Zhejiang University, Hangzhou, 310003 Zhejiang China

**Keywords:** ALPL, Lung adenocarcinoma (LUAD), C-Myc degradation, RhoA, Metastasis

## Abstract

**Background:**

Lung adenocarcinoma (LUAD) metastasis significantly reduces patient survival; hence inhibiting the metastatic ability of lung cancer cells will greatly prolong patient survival. Alkaline phosphatase (ALPL), a homodimeric cell surface phosphohydrolase, is reported to play a controversial role in prostate cancer and ovarian cancer cell migration; however, the function of ALPL in LUAD and the related mechanisms remain unclear.

**Methods:**

TCGA database was used to analysis the expression of ALPL, and further verification was performed in a cohort of 36 LUAD samples by qPCR and western blot. Soft-agar assay, transwell assay and lung metastasis assay were employed to detect the function of ALPL in LUAD progression. The qPCR, luciferase promoter reporter assay and western blot were used to clarify the molecular mechanisms of ALPL in promoting metastasis in LUAD.

**Results:**

ALPL was downregulated in LUAD, and the disease-free survival rate of patients with low ALPL was significantly reduced. Further studies showed that overexpression of ALPL in LUAD cell lines did not significantly affect cell proliferation, but it did significantly attenuate lung metastasis in a mouse model. ALPL downregulation in LUAD led to a decrease in the amount of phosphorylated (p)-ERK. Because p-ERK promotes the classical c-Myc degradation pathway, the decrease in p-ERK led to the accumulation of c-Myc and therefore to an increase in *RhoA* transcription, which enhanced LUAD cell metastasis.

**Conclusion:**

ALPL specially inhibits the metastasis of LUAD cells by affecting the p-ERK/c-Myc/RhoA axis, providing a theoretical basis for the targeted therapy of clinical LUAD.

**Supplementary Information:**

The online version contains supplementary material available at 10.1186/s12935-021-01919-7.

## Background

According to the Global Cancer Analysis Data for 2020, lung cancer accounts for 11.4% of all cancers and has an overall mortality rate of 18.0%. Its morbidity and mortality rates are the highest of the major cancers [1]. Lung cancer is classified as small cell lung cancer (SCLC) or non-small cell lung cancer (NSCLC) according to the tissue type [2]. NSCLC comprises three main subtypes, lung adenocarcinoma (LUAD), lung squamous cell carcinoma (LUSC), and large cell carcinoma. LUAD accounts for approximately 40–50% of NSCLC cases and is thus the main pathological type of lung cancer [3]. Clinically, more than 60% of patients present with later-stage (stage III or IV) disease, which contributes to the poor prognosis of LUAD [3]. Furthermore, the data for 2007–2013 in the USA showed that the 5 year survival rate for lung cancer decreases from 56 to 29% to 5% as the disease progresses from localized to regional and then to distant, respectively [4], indicating that lung cancer metastasis significantly reduces patient survival. Therefore, inhibiting the metastatic ability of lung cancer cells will greatly prolong patient survival.

Studies of the gene encoding alkaline phosphatase (ALPL) are focused mainly on hypophosphatemia. Various mutations in this gene have been shown to affect its activity and cause a decrease in serum ALPL levels, resulting in hypercalcemia and hypokalemia [5]. ALPL plays a crucial role in intracellular signaling and regulation of protein activity [6–8]. Dysfunction of this enzyme is related to the development of multiple diseases, including diabetes mellitus, liver disease, stork and Alzheimer’s disease [9–12]. According to recent reports, ALPL also plays a role in cancer. Rao et al*.* found that knockdown of ALPL can reduce prostate cancer cell migration [10]. Another study suggested that overexpression of ALPL can inhibit the migration and invasion of HGSOC ovarian cancer cells [13]. Therefore, the function of ALPL in cancer and the related mechanisms remain controversial.

c-Myc is one of the most well-characterized transcription factors in tumors. Analysis of a hybrid of rodent and human Burkitt lymphoma cells revealed the presence of a reciprocal translocation between chromosomes 8 and 14 that disrupted the *MYC* gene [14], which confirmed that *MYC* functions as an oncogene in humans [15]. Translocations in *MYC* occur often in hematopoietic cancers, and it was reported to be the third most frequently amplified gene in human cancers in a whole-genome copy number analysis [16, 17]. Its downstream effector, RhoA, is a member of the Rho family of small GTPases and is a key regulator of actin polymerization and cell migration [18, 19]. It acts as a molecular switch in cellular processes such as cell morphogenesis, adhesion, and migration [20, 21]. The primary function of RhoA is to remodel the actin cytoskeleton during cellular processes such as migration and endocytosis, which also affects the local dynamics of microtubules (MTs) [22]. Thus, RhoA clearly plays an important role in cell migration and invasion [23, 24].

In this study, we revealed a molecular mechanism through which ALPL regulates LUAD invasion and metastasis by affecting the c-Myc/RhoA cascade. These findings provide a theoretical basis for the targeted therapy of clinical LUAD.

## Methods

### Plasmids and antibodies

The Plvx-GFP plasmid was purchased from Sunny Biotechnology (Shanghai, China). The Plvx-GFP-ALPL expression plasmid was constructed using the following primers: forward: 5′-CCG CTC GAG GCC ACC ATG ATT TCA CCA TTC TTA GTA CTG GCC -3′, reverse: 5′-CCG GAA TTC GGT GAA CAG GAC GCT CAG GGG-3′. The Plvx-GFP-RhoA expression plasmid was constructed using the following primers: forward: 5′-CCG CTC GAG GCC ACC ATG GCT GCC ATC CGG AAG A-3′, reverse: 5′-CCG GAA TTC ACC CAA GAC AAG GCA CCC AGA T-3′. The RhoA promoter-driven luciferase reporter was constructed into PGL3 Basic vector using primers, forward: 5′-CGG GGT ACC ACT TCC TGT ATC CTG TTG TTT GTG T -3′, reverse: 5′-CCC AAG CTT CAA ATG ACA ATG ACA CAG GAC ATA C -3′. The c-Myc plasmid was from our research group [25]. The antibody against ALPL (GTTX100817) was purchased from Genetex (Irvine, CA, USA). The antibodies against RhoA (2117S), ERK1/2 (4695S), and p-ERK1/2 (4370S) were purchased from Cell Signaling Technology (Beverly, MA, USA). The antibodies against c-Myc (sc-764), RhoA (sc-418) and p-c-Myc (sc-377552) were purchased from Santa Cruz Biotechnology (Santa Cruz, CA, USA). The antibody against GAPDH (GTX100118) was purchased from Genetex. 10X Cell Lysis Buffer (9803) was purchased from Cell Signaling Technology.

### Cell culture and transfection

Normal bronchus epithelial cell line Beas-2B and lung cancer cell lines A549, H1975, HCC827, H1299, and SK-MES-1 were purchased from the American Type Culture Collection (ATCC; Manassas, VA, USA). All cells were subjected to DNA analysis and authenticated before use in these studies [26]. A549 and SK-MES-1 cells were cultured in F12K (21127-022; Gibco/ThermoFisher Scientific, Waltham, MA, USA) supplemented with 10% fetal bovine serum (FBS) (10437-028; Gibco). H1975 cells were cultured in a 1:1 mixture of DMEM/Ham’s F12 medium (10565-018; Gibco) supplemented with 5% FBS. HCC827 and H1299 cells were cultured in RPMI 1640 (11875-093; Gibco) supplemented with 10% FBS, and Beas-2B cells were cultured in DMEM (11995-065; Gibco) supplemented with 10% FBS. Cell transfections were performed using PolyJet DNA In Vitro Transfection Reagent (SignaGen Laboratories, Rockville, MD, USA) according to the manufacturer's instructions. For stable cell line selection, cells were treated with G418 (500–1000 μg/mL) or puromycin (0.2–0.3 μg/mL) depending on the antibiotic resistance plasmid used. Cells surviving the antibiotic selection were pooled as stable mass transfectants [27].

### Reverse transcription-PCR

Total RNA was extracted using TRIzol reagent (Invitrogen/ThermoFisher Scientific) according to the manufacturer’s instructions. Next, 5 μg total RNA was used for first-strand cDNA synthesis with the SuperScript First-Strand Synthesis system and oligo (dT) primers (Invitrogen), as described previously [28].

### Quantitative RT-PCR

Fast SYBR Green Master Mix (4385614; Applied Biosystems/ThermoFisher Scientific) was used for real-time PCR with the 7900HT Fast Real-Time PCR system (Applied Biosystems) [29]. The primers used in this study were as follows: human ALPL (forward: 5′- AC CGA GAT ACA AGC ACT CCC ACT-3′, reverse: 5′- CC GTC ACG TTG TTC CTG TTC AG 3′), human RhoA (forward: 5′-ACT ATG TGG CAG ATA TCG AGG TGG A 3′, reverse: 5′- TA TCA GGG CTG TCG ATG GAA AAA C-3′), human c-Myc (forward: 5′-GGA GGA ACA AGA AGA TGA GGA AGA AA -3′, reverse: 5′-TGA GGA CCA GTG GGC TGT GAG G-3′), and human GAPDH (forward: 5′-GAC TCA TGA CCA CAG TCC ATG C-3′, reverse: 5′-CAG GTC AGG TCC ACC ACT GA-3′).

### Western blot analysis

Whole cell extracts were prepared in cell lysis buffer [10 mM Tris–HCl (pH 7.4), 1% SDS, and 1 mM Na_3_VO_4_]. Western blotting was performed as described previously [27, 30]. Briefly, blots were incubated with primary antibodies diluted 1:500–1:1000 in 5% BSA for 12–16 h at 4 °C, and then incubated with secondary antibody diluted 1:1000–1:2000 in 5% skim milk for 2–3 h at 4 °C. The images were acquired by scanning with the Phosphoimager Typhoon FLA 7000 (GE, Pittsburgh, PA, USA) [27].

### Soft-agar assay

The anchorage-independent growth ability of the cells was evaluated in soft agar, as described [30]. Briefly, 3 mL of 0.5% agar (214010; Becton Dickinson) in basal modified Eagle’s medium supplemented with 10% FBS was layered onto each well of 6-well tissue culture plates. Cells (3 × 10^4^) suspended in 1 mL normal medium (B9638; Sigma-Aldrich) were mixed with 2 mL of 0.5% agar in basal modified Eagle’s medium supplemented with 10% FBS, and 1 mL of the mixture was added into each well on top of the 0.5% agar layer. Plates were incubated at 37 °C in 5% CO_2_ for 3 weeks, and the colonies were counted. The data is presented as the number of colonies/10^4^ cells [30].

### Cell migration and invasion assay

Cell migration and invasion were evaluated using kits from BD Falcon. The assay was performed according to the manufacturer’s instructions, as described previously [27, 31]. Briefly, cells were seeded in the inserts in 400 μL serum-free medium. The inserts were placed in wells containing 700 μL medium with 10% FBS and incubated for 24 h. Then, cells on the upper surface of the filters were removed with a cotton swab. The inserts were fixed in methanol and stained with Giemsa, imaged with a microscope, and then the cells were counted. The data shown are representative of three independent experiments [32].

### Tube formation assay

Tube formation assay was conducted on HUVECs under the treatment of different LUAD cancer cell culture medium. HUVECs (2.0 × 10^4^ cells) were cultured in the presence of the conditioned medium in 96-well plates coated with Matrigel (50 μL/well, BD Biosciences). After 4 h incubation at 37 °C, tube formation was visualized under light microscope. Capillary length was calculated with Image J software. This assay was carried out in triplicate.

### Lung metastasis assay

Female euthymic (nu+ /nu+) mice were purchased from Shanghai Silaike Experimental Animal Company (license no. SCXK, Shanghai 2010 0002; Shanghai, China). At age 4–5 weeks, the mice were randomized and injected with cells [A549 (vector), A549 (ALPL), HCC827 (vector), and HCC827 (ALPL)] via the lateral tail vein (3 × 10^6^ cells in 100 μL PBS/mouse). Detailed experimental methods have been described previously [27].

### Luciferase promoter reporter assay

The RhoA luciferase reporter and pRL-TK were each transiently co-transfected into A549 and HCC827 cells. Luciferase activity was determined 24 h after transfection using the Luciferase Assay System (Promega, Madison, WI, USA), as described [27, 33, 34]. The values were normalized to the internal thymidine kinase (TK) signal. All experiments were conducted in triplicate, and the results were expressed as the mean ± standard error [27].

### H&E staining

Fixed lung tissues were dehydrated using an alcohol gradient, paraffin-embedded, and then stained with H&E, as described in detail previously [35]. Images were captured using the Nikon Eclipse Ni microsystem (Nikon DS-Ri2; Tokyo, Japan). 

### Immunohistochemistry (IHC)

IHC assays were performed to detect the expression of ALPL, p-ERK, c-Myc and RhoA in formalin-fixed paraffin-embedded metastatic lung tumor tissues of mice injected with LUAD cells. Specific antibodies against ALPL (GTTX100817, Genetex), p-ERK1/2 (4370S, CST), c-Myc (sc-764, Santa Cruz) and RhoA (sc-418, Santa Cruz) were used for IHC staining, which were performed using a kit from Boster Bio-Engineering Company (SA1022; Wuhan, China).  Immunostained images were captured with the Nikon Eclipse Ni microsystem (DS-Ri2) and analyzed with Image-Pro Plus version 6.0 (Media Cybernetics, Rockville, MD, USA) by calculating the integrated optical density (IOD) of each stained area (IOD/area). At least five images per specimen were counted.

### Clinical specimens

In total, 36 pairs of human LUAD tissues and corresponding adjacent normal tissues were obtained. The authorized case information is shown in Additional file 1: Table S1. Preoperative biopsy and postoperative pathological examination of the above patients confirmed LUAD. The tissue samples were snap-frozen in liquid nitrogen at the time of surgery, RNA was extracted, and cDNA was synthesized and stored at − 80 °C.

### Statistical analysis

All experimental data are expressed as the mean ± standard deviation (mean ± SD). Graphs and statistical analyses were performed using GraphPad Prism 6 (GraphPad Software, San Diego, CA, USA). The Kaplan–Meier method was used to draw the survival curve, and the log-rank test was used to compare the differences between groups. Comparisons between the control group and the experimental group were performed using the Student’s t-test. Correlation between two independent groups were performed using the Pearson’s Chi-square test. *P* < 0.05 was considered statistically significant.

## Results

### ALPL is downregulated in LUAD and related to patient prognosis

**Fig. 1:**
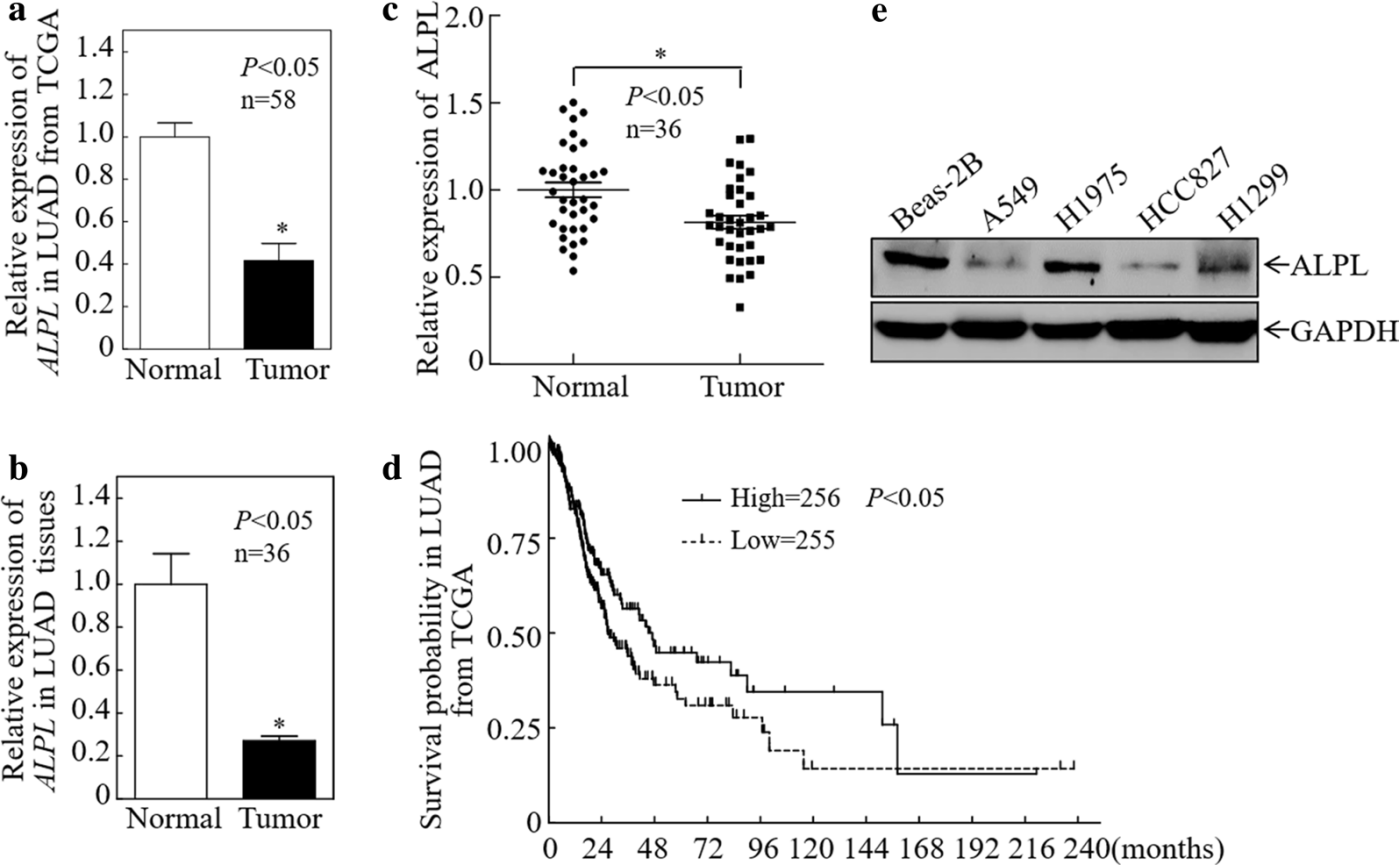
Alkaline phosphatase (ALPL) is downregulated in LUAD. **a** ALPL mRNA expression was analyzed in the 58 paired LUAD cases in the Cancer Genome Atlas (TCGA). **b** ALPL mRNA expression was analyzed in 36 paired fresh clinical LUAD tissues. **c** Western blot analysis of total protein lysates prepared from 36 paired fresh clinical LUAD tissues. Then perform grayscale analysis and statistics. **d** The relationship between ALPL expression in LUAD patients in the TCGA database and the disease-free survival rate. **e** Western blot analysis of ALPL protein levels in LUAD cell lines. GAPDH was used as an internal control. All results were presented as the mean ± SD and analyzed by Student’s *t*-test. The survival curve was analyzed by log-rank test. Asterisks (*) represent statistical significance (*P* < 0.05)

Analysis of the 58 paired LUAD cases in the Cancer Genome Atlas (TCGA) database revealed significantly lower expression of ALPL mRNA in LAUD tumors compared with matched healthy lung tissues (Fig. 1a). A separate qPCR analysis of 36 fresh LUAD samples and matched healthy tissues revealed significantly lower ALPL expression in tumor tissues, consistent with the results obtained using the TCGA database (Fig. 1b). Furthermore, western blot experiments were performed on 36 paired LUAD samples, and grayscale analysis was used to make statistics. The results showed that compared with matched healthy tissues, the expression of ALPL protein in LUAD tumors was significantly down-regulated (Fig. 1c, Additional file 1: Fig. S1). Most important, a survival analysis of LUAD patients in the TCGA database revealed that patients with low expression of ALPL had a significantly worse prognosis (Fig. 1d). We therefore concluded that ALPL downregulation in LUAD might be an important molecular event for cancer development or progression. Then we examined the protein expression of ALPL in normal lung cells and LUAD cells, and the results showed that the expression of ALPL in LUAD cells was lower than that in normal lung cells (Fig. 1e).

### ALPL has no effect on LUAD cell proliferation

**Fig. 2:**
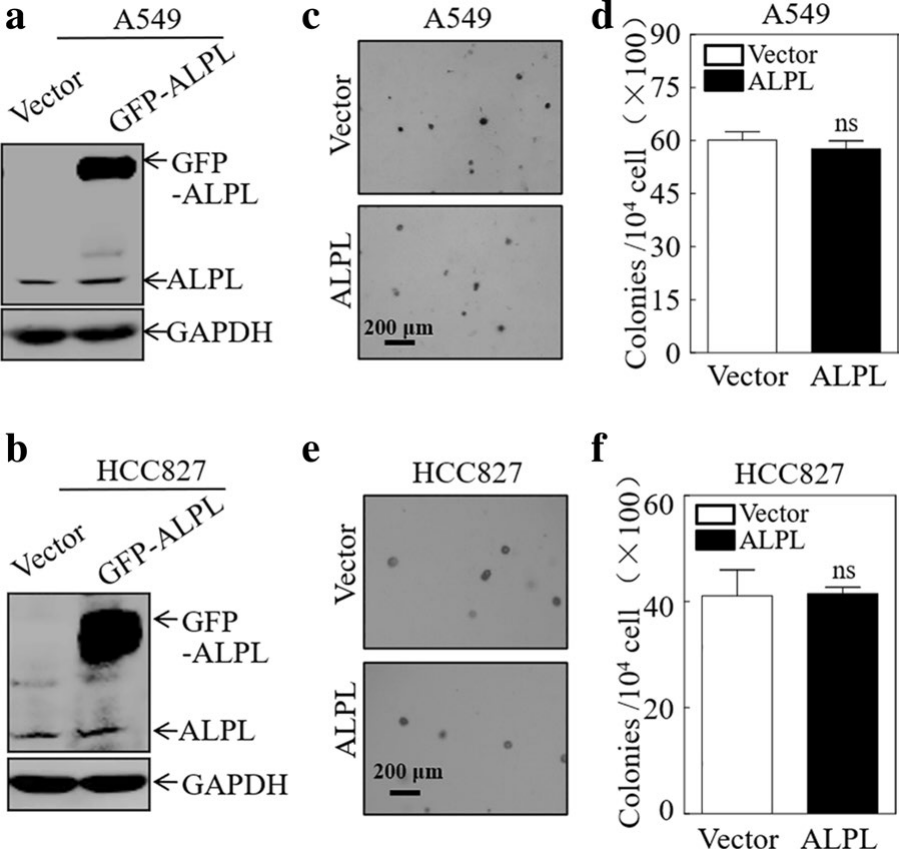
Alkaline phosphatase (ALPL) has no effect on the proliferation of LUAD cells. Western blot analysis of A549 (**a**) and HCC827 (**b**) cells stably transfected with an ALPL overexpression plasmid or a control plasmid. GAPDH was used as an internal control. Soft-agar assay to determine the effect of ALPL overexpression on anchorage-independent growth of A549 (**c**, **d**) and HCC827 (**e**, **f**) cells. Representative images of colonies were captured by microscopy after 3 weeks of incubation (**c**, **e**), Scale bars: 200 µm. The number of colonies with > 32 cells were counted in A549 (Vector) and A549 (ALPL) cells (**d**), and in HCC827 (Vector) and HCC827 (ALPL) cells (**f**). The results represent the number of colonies per 10,000 cells. Data were presented as the mean ± SD and analyzed by Student′s *t*-test, n = 3. ns, no statistical significance relative to the vector control cells (*P* > 0.05)

To investigate the function of ALPL in LUAD cells, we selected two LUAD cell lines, A549 and HCC827, which have relatively low expression of ALPL, and established cell lines stably overexpressing ALPL (Fig. 2a, b). A human lung squamous cell carcinoma (LUSC) cell line (SK-MES-1) stably overexpressing ALPL was also generated (Additional file 1: Fig. S2a). To assess the effects of ALPL on LUAD cell growth, soft-agar experiments were carried out. ALPL overexpression had no effect on the anchorage-independent growth of LUAD (Fig. 2c–f) or LUSC cells (Additional file 1: Fig. S2b, c).

### ALPL significantly inhibits the migration and invasion of LUAD cells, and represses LUAD metastasis in a xenograft model

**Fig. 3:**
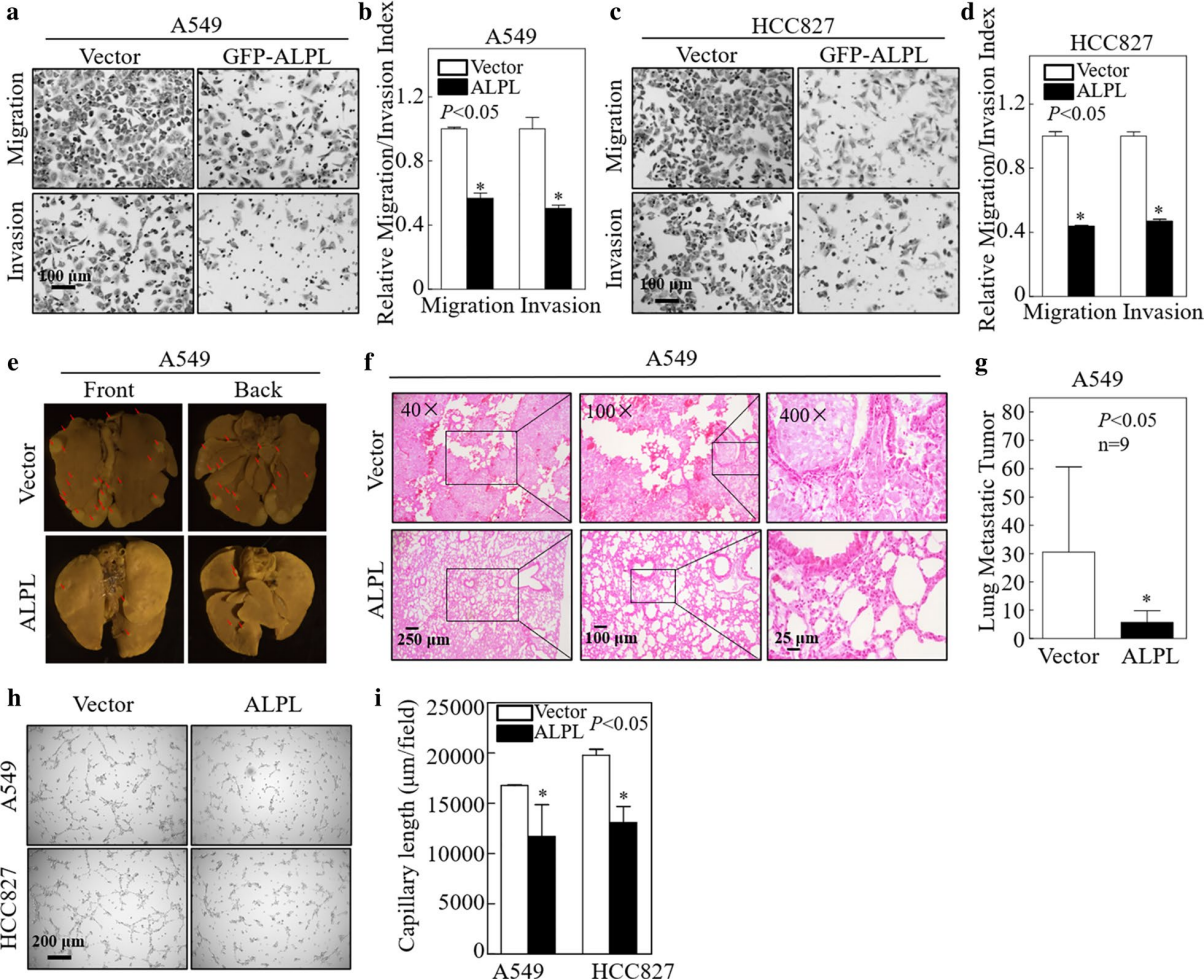
Alkaline phosphatase (ALPL) inhibited the metastasis of LUAD cells in vivo and in vitro. Transwell assays were performed to determine the effect of ALPL overexpression on A549 (**a**) and HCC827 (**c**) cells, Scale bars: 100 µm. **b**, **d** The migration and invasion rate of A549 (**a**) and HCC827 (**c**) cells, n = 3. **e** A tail vein injection model was used to evaluate lung metastasis. The front and back images of the lungs are shown, n = 9. **f** Representative H&E images of A549 (Vector) and A549 (ALPL) tumors, Scale bars: 250 µm, 100 µm or 25 µm. **g** Quantification of the number of metastatic lung tumors in five pairs of nude mice. **h** Tube formation assay were performed to determine the effect of ALPL overexpression on the angiogenesis activity of A549 and HCC827, Scale bars: 100 µm. **i** Capillary length was calculated in A549 and HCC827 cell culture medium. Data were presented as the mean ± SD and analyzed by Student′s *t*-test. Asterisks (*) represent statistical significance (*P* < 0.05)

The prognosis of lung cancer is significantly worse once it has metastasized. Evaluation of the TCGA database revealed higher levels of *ALPL* in patients with stage I LAUD compared with patients with later-stage disease (II, III, and IV; Additional file 1: Fig. S3). This suggested that ALPL expression may be closely related to LUAD metastasis. To evaluate this, we performed Transwell and wound healing experiments to investigate the effects of ALPL on LUAD cell migration and invasion. ALPL overexpression significantly inhibited the migration and invasion of both A549 and HCC827 cells (Fig. 3a–d, Additional file 1: Fig. S4). By contrast, ALPL overexpression had no effect on the migration or invasion of LUSC cells (SK-MES-1; Additional file 1: Fig. S2d, e). This result may explain the observation that the survival of LUSC patients in the TCGA database was not related to ALPL expression (Additional file 1: Fig. S5). The effect of ALPL on the metastatic capacity of LUAD cells was verified in vivo in a nude mouse xenograft model of lung cancer metastasis. ALPL overexpression significantly reduced the number and size of A549 lung cancer metastases (Fig. 3e–g, Additional file 1: Fig. S6). ALPL overexpression had the same effect in HCC827 cells (Additional file 1: Fig. S7a–c). Moreover, the results of tube formation assay showed that ALPL overexpression also inhibited angiogenesis (Fig. 3h, i). Therefore, in both in vivo and in vitro experiments, ALPL overexpression specially inhibited the migration of LUAD cells.

### ALPL inhibits LUAD cell migration by downregulating RhoA

**Fig. 4:**
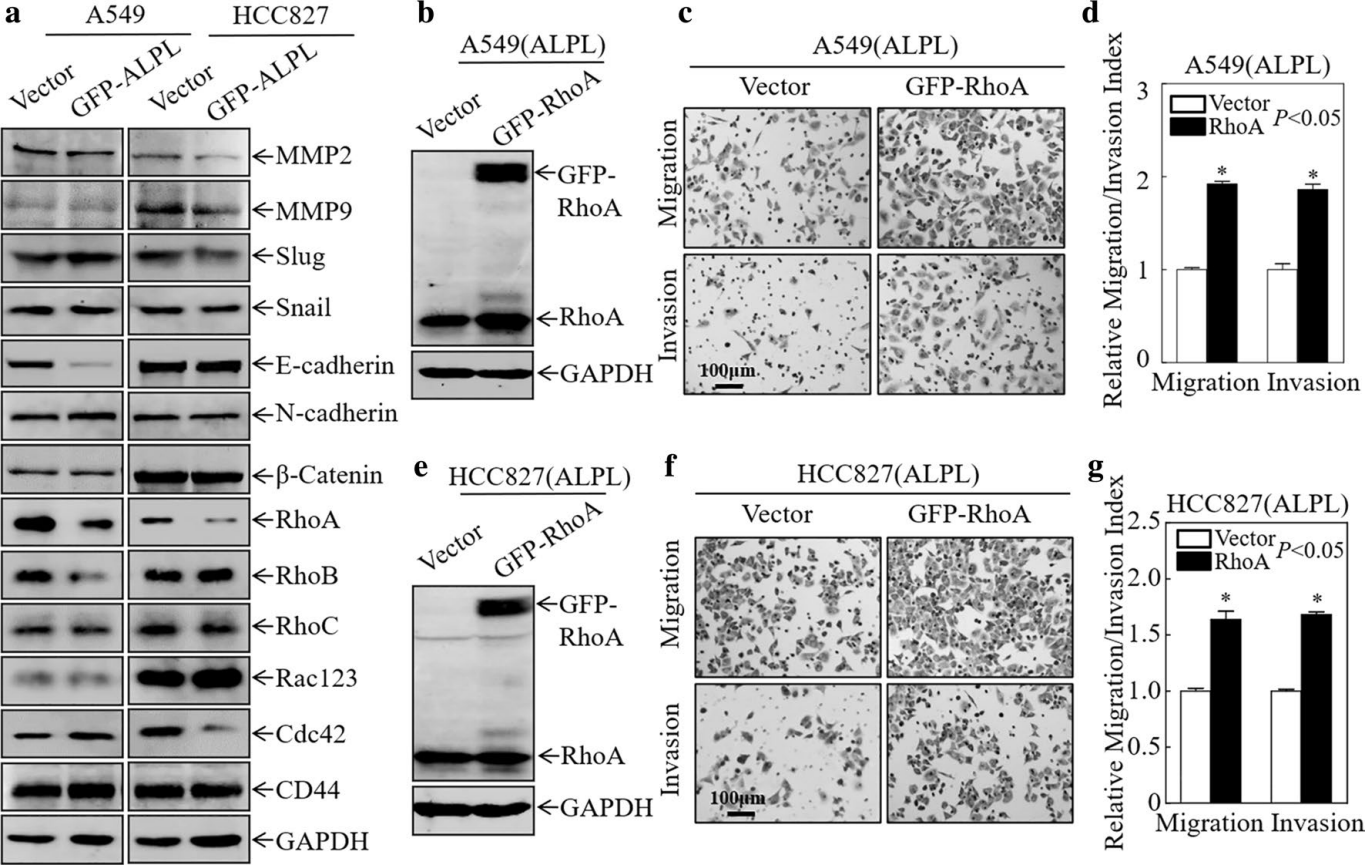
Alkaline phosphatase (ALPL) inhibits the migration and invasion of LUAD cells by downregulating RhoA protein expression. **a** Western blot analysis of cell lysates from the indicated cells. **b**, **e** Western blot analysis of GFP-RhoA and RhoA expression in A549 (ALPL) and HCC827 (ALPL) cells stably transfected with GFP-RhoA or a vector control plasmid. GAPDH was used as a loading control. Transwell assays were performed to determine the effect of RhoA overexpression on A549 (ALPL) (**c**) and HCC827 (ALPL) (**f**) cells, Scale bars: 100 µm. **d**, **g** The migration and invasion rate of A549 (ALPL) and HCC827 (ALPL) cells. Data were presented as the mean ± SD and analyzed by Student′s *t*-test, n = 3. Asterisks (*) indicate a significant increase relative to control vector cells (*P* < 0.05)

To elucidate the molecular mechanism through which ALPL inhibits LUAD cell migration and invasion, western blot assays were performed to evaluate the expression of molecules related to cell motility. Matrix metalloproteinase (MMP)2 and MMP9 enhance the invasion of cancer cells by degrading type IV collagen [36, 37]. Slug, Snail, E-cadherin, N-cadherin, and β-catenin, which are involved in epithelial-mesenchymal transition (EMT), have also been reported to promote cancer cell migration and invasion [17, 18, 38–43], while RhoA, Cdc42, and Rac1 are members of the Rho family of small GTPases, and are key mediators of actin polymerization and cell migration. These molecules play active roles in cell migration and invasion [22, 44, 45]. RhoA was consistently and significantly downregulated in A549 and HCC827 cells after overexpression of ALPL, while the other molecules were not consistently affected by ALPL expression (Fig. 4a). Therefore, we hypothesized that ALPL inhibited LUAD cell migration by downregulating RhoA expression. To validate this hypothesis, RhoA was stably overexpressed in the ALPL-overexpressing cell lines [A549 (ALPL) and HCC827 (ALPL)], as shown in Fig. 4b and e. Overexpression of RhoA restored the migratory ability of A549 and HCC827 transfectants (Fig. 4c, d and f, g). These findings suggest that ALPL inhibits LUAD cell migration by downregulating RhoA.

### ALPL inhibits *RhoA* transcription by downregulating c-Myc

**Fig. 5:**
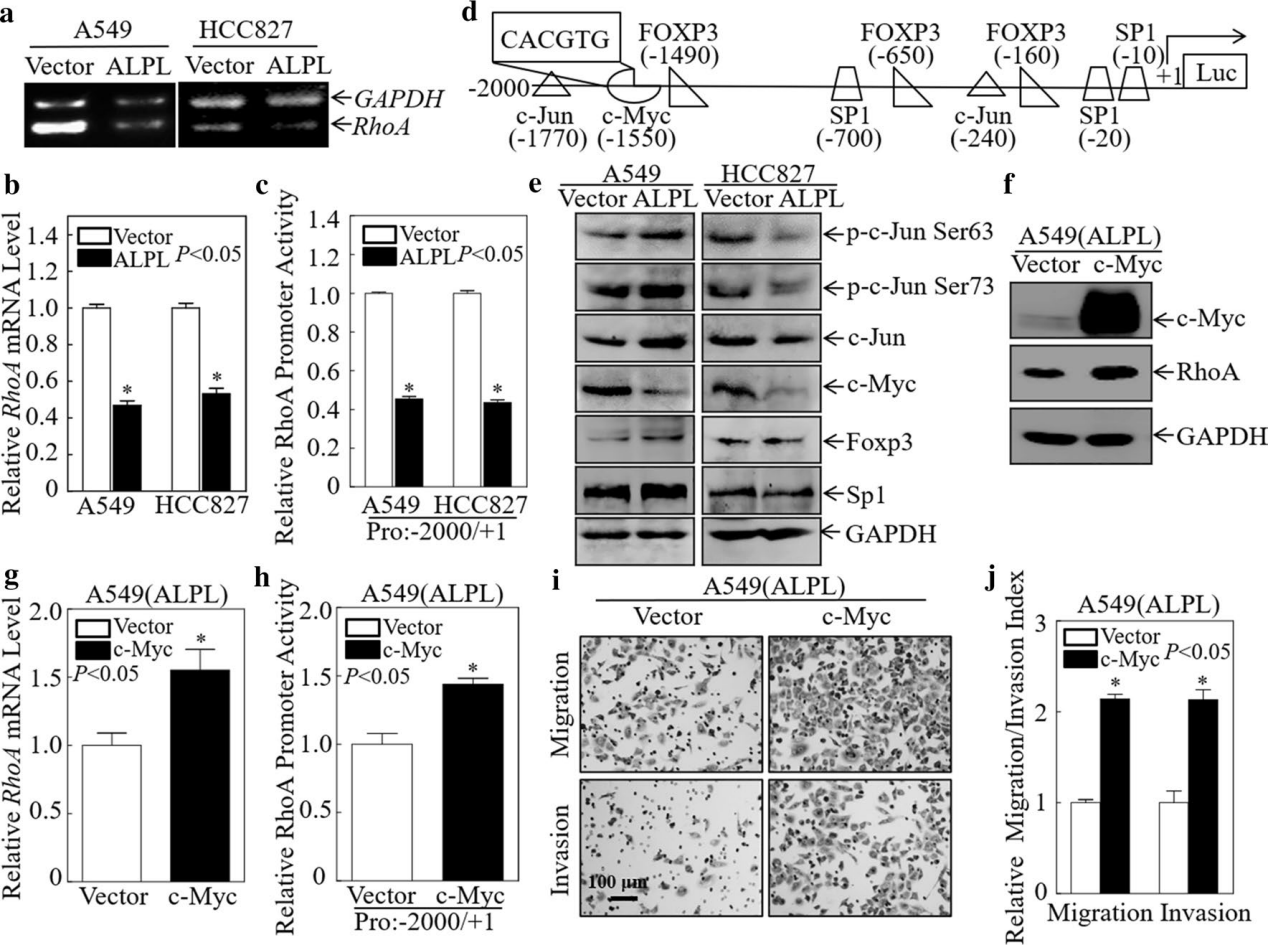
Alkaline phosphatase (ALPL) inhibits RhoA mRNA transcription by downregulating c-Myc. **a** Semi-quantitative PCR was used to evaluate RhoA mRNA expression after ALPL overexpression in A549 and HCC827 cells. **b** RhoA mRNA expression was evaluated by qPCR after ALPL overexpression in A549 and HCC827 cells. **c** Dual-luciferase reporter assays were used to detect RhoA promoter activity after ALPL overexpression in A549 and HCC827 cells. **d** TFANSFAC Transcription Factor Binding Sites Software (Biological Database, Wolfenbuttel, Germany) was used for bioinformatics analysis of the RhoA promoter region. Transcription factors for which potentially conserved binding sites were identified in the RhoA promoter region included c-Myc, c-Jun, Foxp3, and Sp1. **e** Western blot analysis of cell lysates from the indicated cells. GAPDH was used as an internal control. **f** Western blot analysis of c-Myc and RhoA expression in A549 (ALPL) cells stably transfected with c-Myc or a control plasmid. GAPDH was used as a loading control. **g** The RhoA mRNA expression level was evaluated by qPCR after overexpression of c-Myc in A549 (ALPL) cells. **h** Dual-luciferase reporter assays of the *RhoA* promoter activity after overexpression of c-Myc in A549 (ALPL) cells. **i** Transwell assays to determine the effect of overexpression of c-Myc on A549 (ALPL) cells, Scale bars: 100 µm. **j** The migration and invasion rate of A549 (ALPL) cells. All results were presented as the mean ± SD and analyzed by Student’s *t*-test, n = 3. Asterisks (*) represent statistical significance (*P* < 0.05)

To further explore the mechanism by which ALPL downregulates RhoA expression, we first conducted a semi-quantitative PCR analysis of the RhoA mRNA level. RhoA mRNA was significantly downregulated in A549 (ALPL) and HCC827 (ALPL) cells by PCR (Fig. 5a) and qPCR (Fig. 5b). These data indicated that ALPL downregulated RhoA expression at the mRNA level. To investigate the mechanism underlying ALPL downregulation of RhoA, we investigated *RhoA* promoter activity by dual-luciferase reporter assays. As shown in Fig. 5c, *RhoA* promoter activity was significantly downregulated in both cell lines after ALPL overexpression, indicating that ALPL downregulates RhoA expression by inhibiting its transcription.

To further investigate the effects of ALPL on *RhoA* transcription, the TFANSFAC Transcription Factor Binding Sites Software (Biological Database, Wolfenbuttel, Germany) was used to predict transcription factors controlling RhoA expression. Transcription factors for which potentially conserved binding sites in the *RhoA* promoter region were identified included c-Myc, c-Jun, Foxp3, and Sp1 (Fig. 5d). We examined the expression of these potential transcription factors in A549 and HCC827 cells by western blot analysis. c-Myc protein levels were significantly downregulated in A549 (ALPL) and HCC827 (ALPL) cells, while the other molecules showed no signs of consistent downregulation (Fig. 5e). It has been reported that c-Myc directly regulates *RhoA* transcription [44]. We hypothesized that c-Myc may be a key molecule in the mechanism through which ALPL regulates *RhoA* transcription. To test this hypothesis, we constructed an A549 (ALPL) stable cell line that expresses c-Myc and found that RhoA protein expression was restored (Fig. 5f). Subsequent qPCR analysis confirmed that RhoA expression was also restored at the mRNA level (Fig. 5g). Dual-luciferase reporter assays also showed that overexpression of c-Myc restored the level of *RhoA* promoter activity in A549 (ALPL) cells (Fig. 5h). These data suggested that ALPL-downregulated c-Myc expression in LUAD resulted in reduced RhoA expression, which in turn resulted in decreased LUAD cell migration and invasion. To further verify this, we performed three independent Transwell experiments following transfection of A549 (ALPL) cells with c-Myc. The migration and invasion ability of A549 (ALPL) cells was restored following transfection with c-Myc (Fig. 5i, j). These data confirm that ALPL downregulates RhoA by inhibiting the expression of c-Myc, resulting in reduced LUAD cell migration and invasion.

### ALPL promotes the degradation of c-Myc by activating ERK

**Fig. 6:**
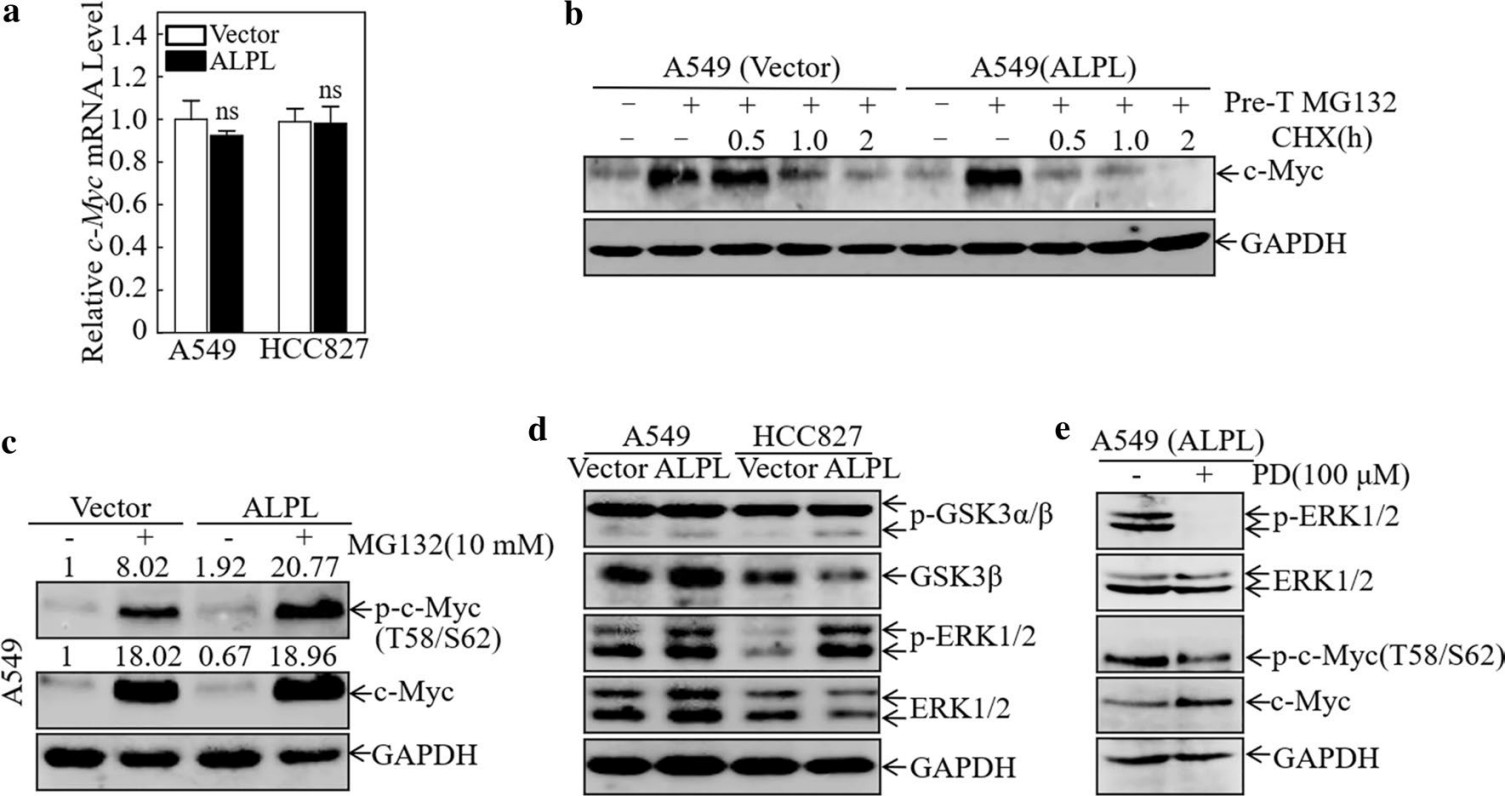
Alkaline phosphatase (ALPL) promotes the degradation of c-Myc by activating ERK. **a** qPCR analysis of the levels of c-Myc mRNA in A549 and HCC827 cells overexpressing ALPL. ns, not significant. **b** The rate of c-Myc protein degradation in A549 (Vector) and A549 (ALPL) cells. GAPDH was used as an internal control. **c** Western blot analysis of the levels of c-Myc and p-c-Myc (Thr58/Ser62) in A549 (Vector) and A549 (ALPL) cells after treatment with MG132. **d** Western blot analysis of the indicated cells for levels of the ubiquitin-related molecules ERK1/2, GSK-3β, p-ERK1/2, and p-GSK-3α/β. GAPDH was used as an internal control. **e** Western blot analysis of the levels of p-ERK1/2, ERK1/2, c-Myc, and p-c-Myc (Thr58/Ser62) in A549 (ALPL) cells after treatment with PD98059 (100 μM)

To further explore the mechanism by which ALPL regulates c-Myc, we analyzed c-Myc mRNA levels by qPCR. There were no significant differences in c-Myc mRNA levels in A549 (ALPL) and HCC827 (ALPL) cells compared with the corresponding wild-type cell lines (Fig. 6a), indicating that ALPL does not regulate c-Myc at the transcriptional level. A variety of proteins have been reported to play roles in cancer development by regulating the degradation of c-Myc protein [45, 46]. Therefore, we evaluated c-Myc protein degradation in control A549 and A549 (ALPL) cells. ALPL overexpression significantly enhanced the degradation rate of c-Myc protein (Fig. 6b), indicating that ALPL downregulates c-Myc expression by enhancing its degradation. It has been reported that degradation of c-Myc usually occurs via classical ubiquitin-dependent degradation pathways, in which ERK- and GSK-3-mediated phosphorylation of c-Myc at Ser62 and Thr58 tags it for degradation [47, 48]. To confirm that ALPL degradation of c-Myc is ubiquitin-dependent, we investigated the effects of the proteasome inhibitor MG132 on c-Myc levels and phosphorylation in control A549 and A549 (ALPL) cells. After treatment with MG132, c-Myc accumulated in both the control A549 and A549 (ALPL) cells, with no significant difference between the two cell lines. However, levels of p–c-Myc (Thr58/Ser62) were much higher in A549 (ALPL) cells than in control cells (Fig. 6c). These results suggested that ALPL accelerated the degradation of c-Myc by promoting its phosphorylation at Thr58 and Ser62, resulting in ubiquitin-dependent degradation.

To determine whether c-Myc phosphorylation at Thr58 and Ser62 was mediated by ERK or GSK-3, we analyzed the expression of ERK1/2, GSK-3β, p-ERK1/2, and p-GSK-3α/β (Ser21/9) in both A549 (ALPL) and HCC827 (ALPL) cells (Fig. 6d). Western blot analysis showed that p-ERK and p-GSK-3β (Ser9) were significantly up-regulated in A549 (ALPL) and HCC827 (ALPL) cells, while the other molecules were not significantly affected by ALPL overexpression. GSK-3 activity is significantly inhibited after phosphorylation at Ser9; therefore, we further speculated that ALPL accelerated the degradation of c-Myc by activating ERK1/2, resulting in the increase in p-c-Myc (Thr58/Ser62). To test this hypothesis, A549 (ALPL) cells were treated with the p-ERK inhibitor PD-98059. Phosphorylation of ERK was almost completely inhibited in the PD-98059-treated cells, while c-Myc expression increased and the p-c-Myc (Thr58/Ser62) level decreased (Fig. 6e). These findings confirmed our hypothesis that ALPL promotes phosphorylation of c-Myc by activating ERK, leading to accelerated c-Myc degradation.

### ALPL overexpression increases p-ERK accompanied with c-Myc and RhoA downregulation in metastatic lung tumor tissues of mouse model

**Fig. 7:**
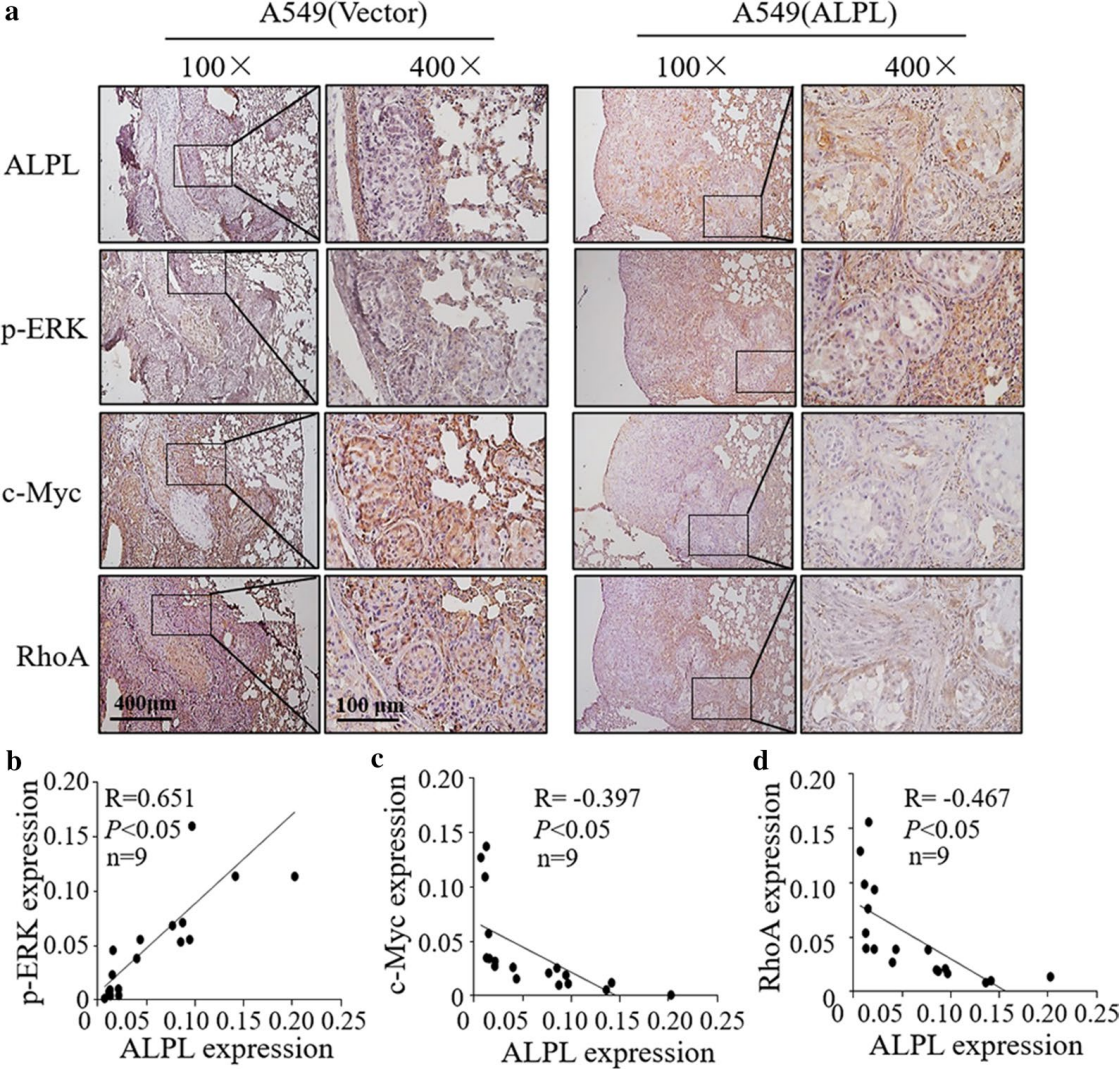
The expression of ALPL, p-ERK, c-Myc and RhoA were determined by IHC staining in the metastatic lung tumors tissues. **a** Representative IHC images showing the expression of ALPL, p-ERK, c-Myc and RhoA in metastatic lung tumors tissues, n = 9, Scale bars: 400 µm or 100 µm. Protein expression levels of p-ERK (**b**), c-Myc (**c**), and RhoA (**d**) were analyzed by calculating the integrated optical density per stained area (IOD/area) using Image-Pro Plus version 6.0, and the correlation between p-ERK/c-Myc/RhoA and ALPL was analyzed by Pearson’s Chi-square test

**Fig. 8:**
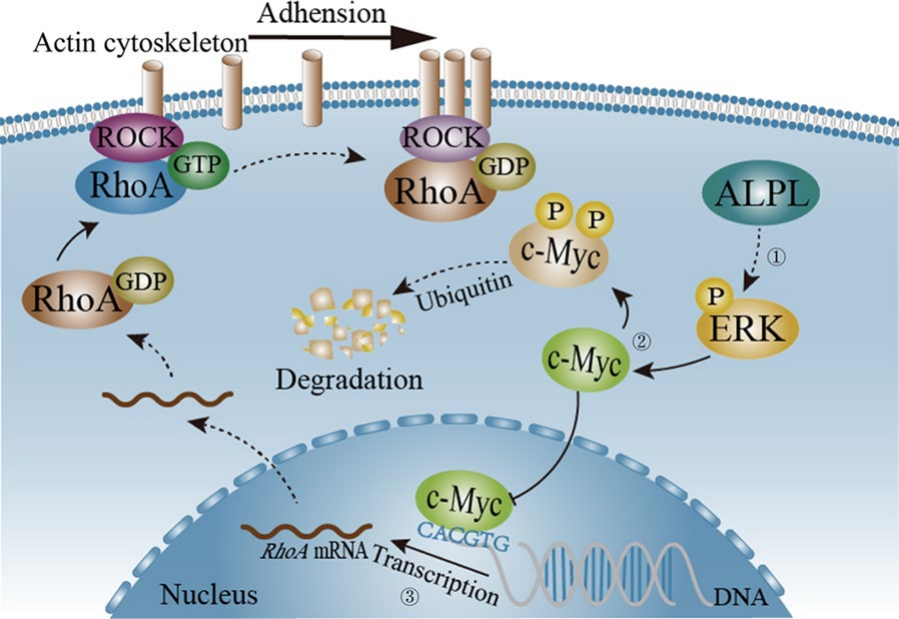
Schematic diagram of the pathway by which ALPL downregulates RhoA via c-Myc. ① ALPL leads to an accumulation of phosphorylated (p)-ERK. ② p-ERK promotes c-Myc phosphorylation at Thr58 and Ser62 which is then degradated through ubiquitin–proteasome system. ③ The reduction of c-Myc leads to a decrease in *RhoA* transcription, which eventually suppresses LUAD cell metastasis. The dotted line indicates a multi-process, and the solid line indicates a single process

To determine whether ALPL exerts a regulatory effect on p-ERK/c-Myc/RhoA in vivo, we assessed the expression of ALPL, p-ERK, c-Myc and RhoA using IHC in metastatic lung tumor tissues of mouse models that are generated with A549 (ALPL) and HCC827 (ALPL) in comparison to A549 (Vector) and HCC827 (Vector), respectively, and found that a remarkable upregulation of p-ERK but c-Myc and RhoA downregulation in metastatic lung tumor tissues of mouse models generated by injection of A549 (ALPL) and HCC827(ALPL) cells (Fig. 7a–d, Additional file 1: Fig. S8a–d). RhoA is positively correlated with c-Myc expression, while c-Myc expression is negatively correlated with p-ERK expression. Collectively, these results demonstrated that ALPL expression downregulated c-Myc by enhancing ERK phosphorylation, leading to a decrease in c-Myc-mediated RhoA protein expression, which eventually suppresses LUAD cell metastasis (Fig. 8).

## Discussion

In this study, we show for the first time that ALPL specially inhibits the metastasis of LUAD cells but not LUSC cells. This not only indicates that ALPL plays a unique role in the pathological development of LUAD, but also implicates ALPL as a specific target for inhibiting the metastasis of LUAD. We also show that the inhibitory effects of ALPL on the metastasis of LUAD cells are mediated via the p-ERK/c-Myc/RhoA axis. ERK, c-Myc and RhoA are the genes playing vital roles in tumor metastasis of many cancers including lung cancer. It is of great significance to develop drugs targeting ERK, c-Myc and RhoA for the treatment and prevention of tumor metastasis. Our results showed that ALPL can significantly inhibit the invasion of LUAD cells in vitro and in vivo, which is mediated via the ALPL/p-ERK/c-Myc/RhoA axis. Therefore, our findings provide a theoretical basis for the development of strategies targeting ALPL for the treatment of LUAD.

ALPL is commonly used to evaluate acute liver damage and cholestasis [49]. Studies have also shown that ALPL is associated with hypophosphatemia [5, 50], skeletal development, cardiovascular disease, and acute kidney injury [51–53]. It has been reported that mutations in ALPL affect its ability to cause disease [5]. We did not identify any mutations in ALPL in LUAD using the TCGA database, suggesting that metastasis of LUAD cells is related to inhibition of ALPL expression rather than mutation. LUAD cells also had lower expression of ALPL than normal lung cells. ALPL also plays very different roles in different tumor types. Reduced expression of tumor-derived ALPL inhibited mesenchymal-to-epithelial transition and the migration of advanced prostate cancer [10]. Conversely, ALPL functions as a tumor suppressor gene in meningioma [54] and ovarian cancer [13]. In this study, we show for the first time that ALPL inhibits the metastasis of LUAD cells via the p-ERK1/2-c-Myc-RhoA axis, which extends our understanding of the role of ALPL in cancer development. Although the expression of ALPL is not tissue-specific, the function of ALPL is influenced by its cellular context. ALPL expression is elevated after acute injury of some organs such as the liver and kidneys [49, 53]. Our finding that downregulation of ALPL promotes metastasis of LUAD cells highlights the diverse functions of ALPL in different settings. We further found that ALPL inhibited metastasis of LUAD cells but did not play a similar role in LUSC cells, and this phenomenon deserves further exploration.

In this study, we demonstrated that ALPL enhances ERK phosphorylation; however, previous studies have shown that ALPL downregulates its targets [6]. Lee et al*.* found that the human dental pulp cells were cultured in osteogenic medium, ALPL and p-ERK were up-regulated, while MKP-1 was downregulated [55]. As a member of the MAP kinase phosphatase (MKP) family, MKP-1 inactivates MAP kinases including ERK1/2 [56], and its degradation can be attenuated by inhibitors of the ubiquitin-directed proteasome complex. We speculate that ALPL may increase ERK phosphorylation by regulating MKP-1, although this remains to be clarified. ERK is a member of the MAPK family and promotes ubiquitin-dependent degradation of c-Myc by phosphorylation [47, 48]. In our study, ALPL increased ERK phosphorylation, resulting in increased phosphorylation of c-Myc, thereby promoting c-Myc degradation. Furthermore, in LUAD cells, c-Myc degradation was reduced because p-ERK was reduced. Previous studies have shown that GSK3 plays a key role in the ubiquitin-dependent degradation of c-Myc [47, 48]. However, our results indicated that p-ERK, not p-GSK3, is the key molecule through which ALPL promotes c-Myc ubiquitination. This finding led us to re-examine the status of p-ERK in the ubiquitin-dependent degradation of c-Myc.

c-Myc promotes RhoA mRNA expression, thereby enhancing the migration and invasion ability of tumor cells [44]. We demonstrate here that ALPL enhances the ubiquitin-dependent degradation of c-Myc by p-ERK. c-Myc is a well-known oncogene [15], and inhibition of c-Myc in cancer is a potential therapeutic strategy. Thus, the ability of ALPL to down-regulate c-Myc may represent a novel approach to cancer treatment. As a member of the Rho family, RhoA plays an important role in cancer cell metastasis [23, 24]. Overexpression of RhoA significantly restored the inhibitory effect of ALPL on LUAD cells metastasis. Our study is the first to show that ALPL reduces the expression of RhoA to inhibit the metastasis of LUAD cells. In summary, ALPL promotes ubiquitin-dependent c-Myc degradation by activating ERK. Reduced c-Myc expression results in reduced RhoA transcription, thereby inhibiting cell migration and invasion. Accordingly, downregulation of ALPL decreased ERK phosphorylation in LUAD, causing an accumulation of c-Myc, which promotes RhoA transcription, leading to increased metastasis of LUAD cells (Fig. 8).

## Conclusions

Overall, we evidenced for the first time that ALPL specially inhibits the metastasis of LUAD cells but not LUSC cells, and revealed a molecular mechanism through which ALPL regulates LUAD invasion and metastasis by affecting the p-ERK/c-Myc/RhoA axis. These results implicated ALPL as a specific target for inhibiting the metastasis of LUAD and provided a theoretical basis for the targeted therapy of clinical LUAD.

## Supplementary Information

**Additional file 1.**Additional Table and Figures.

## Data Availability

Bioinformatics results announced here based on data produced by the TCGA research network (http://cancergenome.nih.gov/). All data generated or analyzed during this study are included in this published article and its supplementary information files.
