# Supplementary material for: Alkaline phosphatase downregulation promotes lung adenocarcinoma metastasis via the c-Myc/RhoA axis
Source: Cancer Cell Int. 2021 Apr 15;21:217. doi: 10.1186/s12935-021-01919-7 (PMC8050923; doi:10.1186/s12935-021-01919-7)
Supplement: Supplementary file 1 — Additional file 1.Additional Table and Figures. [file 12935_2021_1919_MOESM1_ESM.docx]

**Supplementary Materials**

**Supplementary Table 1.** The information of lung cancer patients including case number, gender, age and tumor type.

| **case**  **(NO#)** | **gender** | **age** | **tumor**  **type** | **case**  **(NO#)** | **gender** | **age** | **tumor**  **type** |
| --- | --- | --- | --- | --- | --- | --- | --- |
| 1# | male | 77 | LUAD | 19# | male | 59 | LUAD |
| 2# | male | 59 | LUAD | 20# | female | 44 | LUAD |
| 3# | male | 63 | LUAD | 21# | male | 62 | LUAD |
| 4# | female | 63 | LUAD | 22# | male | 52 | LUAD |
| 5# | female | 68 | LUAD | 23# | male | 67 | LUAD |
| 6# | female | 66 | LUAD | 24# | female | 74 | LUAD |
| 7# | female | 56 | LUAD | 25# | female | 59 | LUAD |
| 8# | female | 58 | LUAD | 26# | female | 59 | LUAD |
| 9# | female | 54 | LUAD | 27# | female | 58 | LUAD |
| 10# | female | 56 | LUAD | 28# | female | 69 | LUAD |
| 11# | female | 55 | LUAD | 29# | male | 73 | LUAD |
| 12# | male | 67 | LUAD | 30# | female | 62 | LUAD |
| 13# | female | 69 | LUAD | 31# | male | 60 | LUAD |
| 14# | male | 54 | LUAD | 32# | female | 54 | LUAD |
| 15# | female | 66 | LUAD | 33# | female | 61 | LUAD |
| 16# | female | 61 | LUAD | 34# | female | 67 | LUAD |
| 17# | male | 70 | LUAD | 35# | male | 61 | LUAD |
| 18# | male | 49 | LUAD | 36# | female | 74 | LUAD |

**
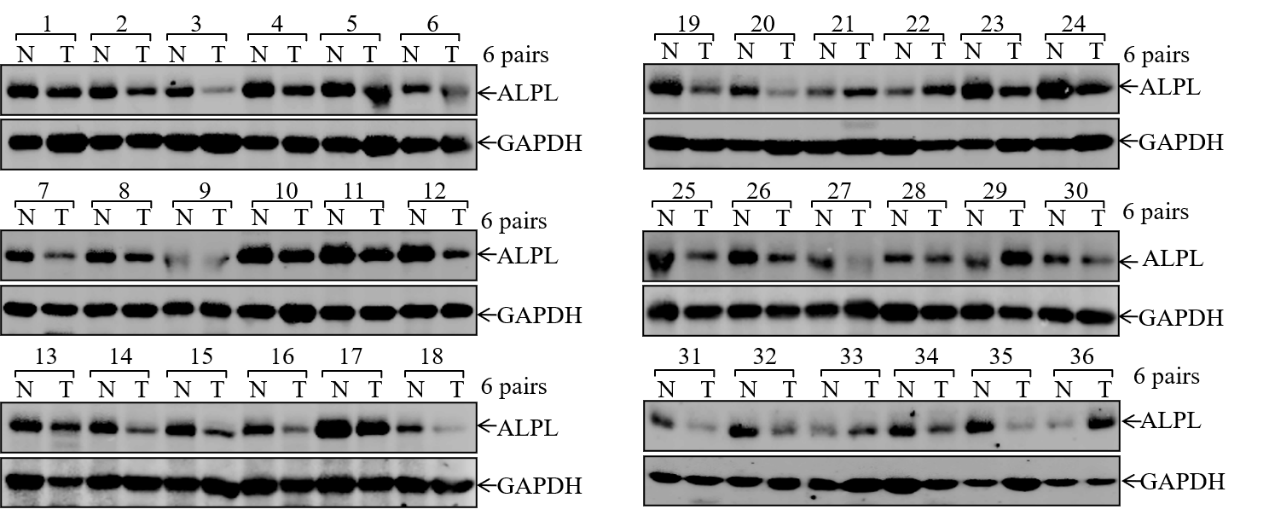
**

**Fig. S1** Western blot analysis of Alkaline phosphatase (ALPL) expression levels in 36 paired fresh clinical LUAD tissues, n=36.

**
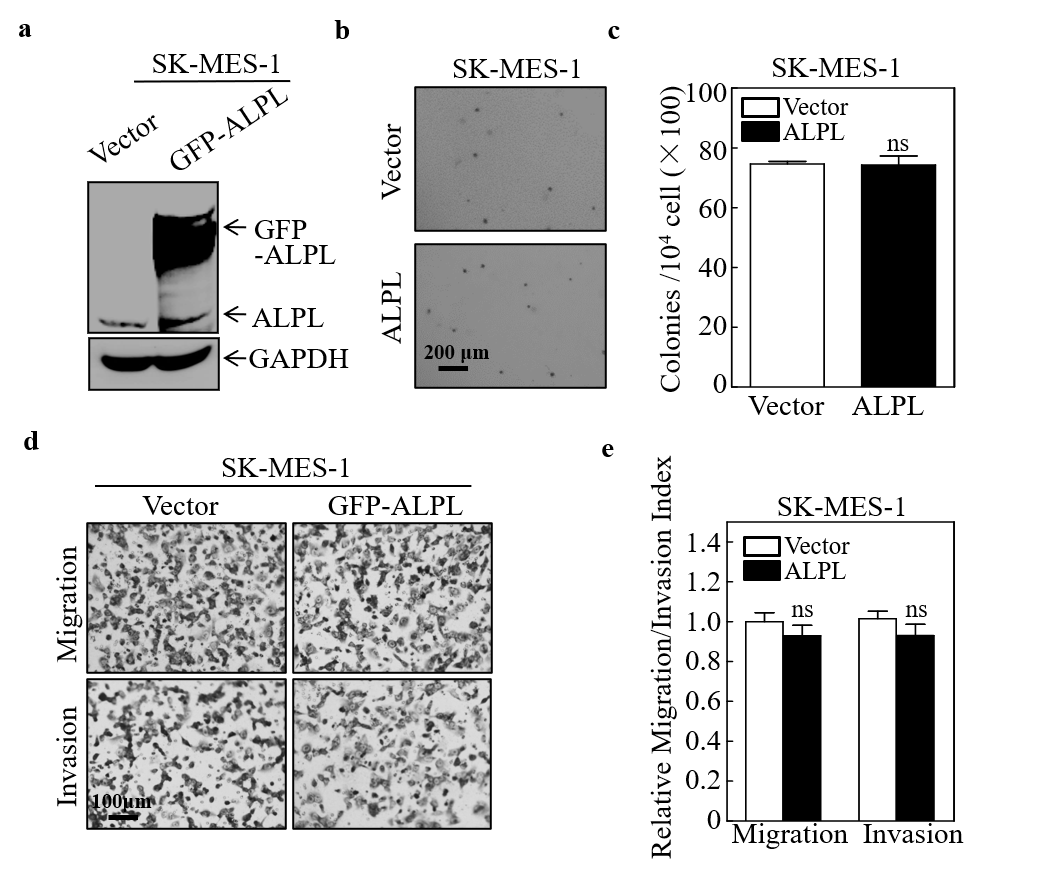
**

**Fig. S2** Alkaline phosphatase (ALPL) has no significant effect on the proliferation or metastasis of LUSC cells. (**a**) Western blot analysis of SK-MES-1 cells stably transfected with an ALPL-expressing or control plasmid. GAPDH was used as an internal control. (**b**) Soft-agar assays to determine the effect of ALPL overexpression on the anchorage-independent growth of SK-MES-1 cells. Representative images of colonies of the indicated cells were captured by microscopy after 3 weeks of incubation. (**c**) SK-MES-1 (Vector) and SK-MES-1 (ALPL) colonies with >32 cells were counted. The results represent the number of colonies per 10,000 cells. ns, no significance relative to the vector control cells (*P*> 0.05). (**d**) Transwell assays to determine the effect of ALPL overexpression on SK-MES-1 cell migration and invasion. (**e**) The migration and invasion rate of SK-MES-1
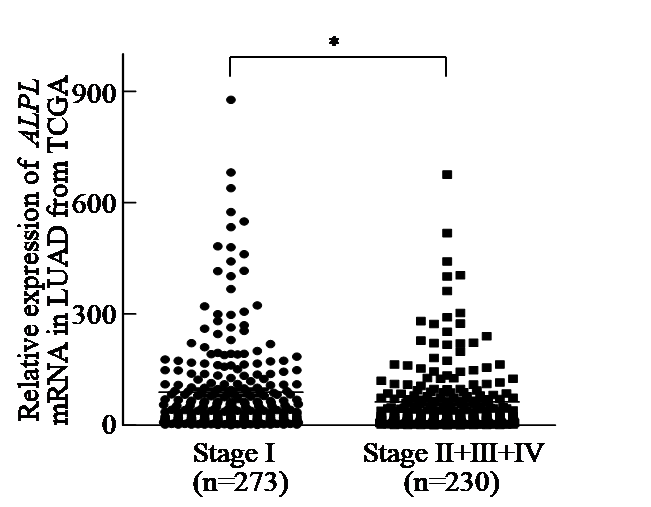
cells. Asterisks (*) indicate a significant increase relative to control vector cells (*P*< 0.05).

**Fig. S3** Patients with stage I LUAD have higher alkaline phosphatase (ALPL) expression than those with stage II, III, and IV disease. ALPL expression according to tumor stage in the TCGA database. Asterisks (*) represent statistical signiﬁcance (*P*<0.05).
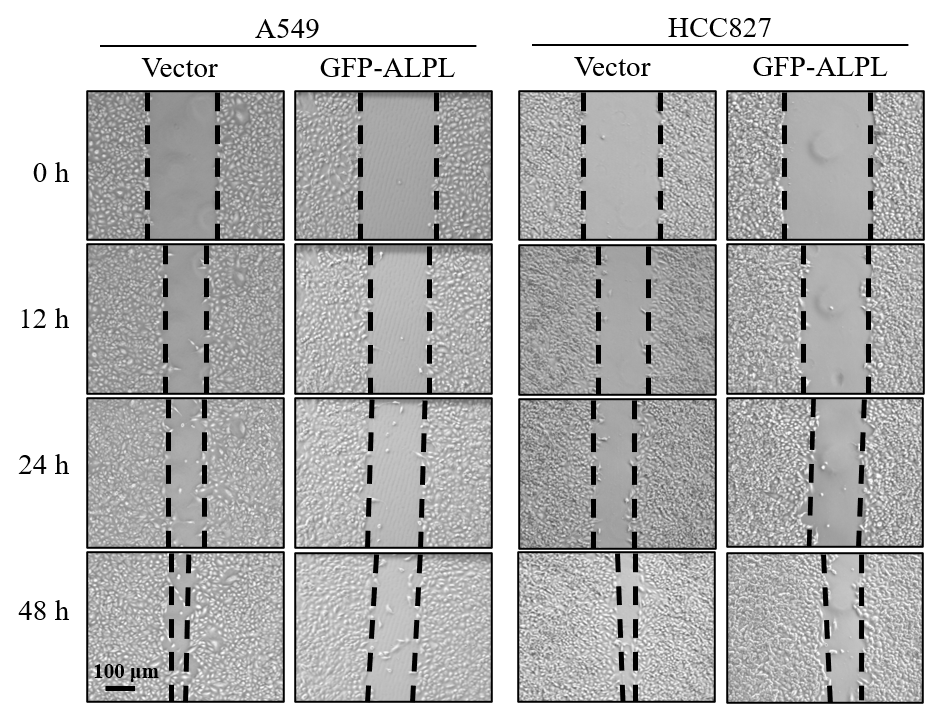


**Fig. S4** Alkaline phosphatase (ALPL) inhibites LUAD cell migration. Wound healing assays were performed to determine the effect of ALPL overexpression on A549 and HCC827 cells migration, Scale bars: 100 µm.


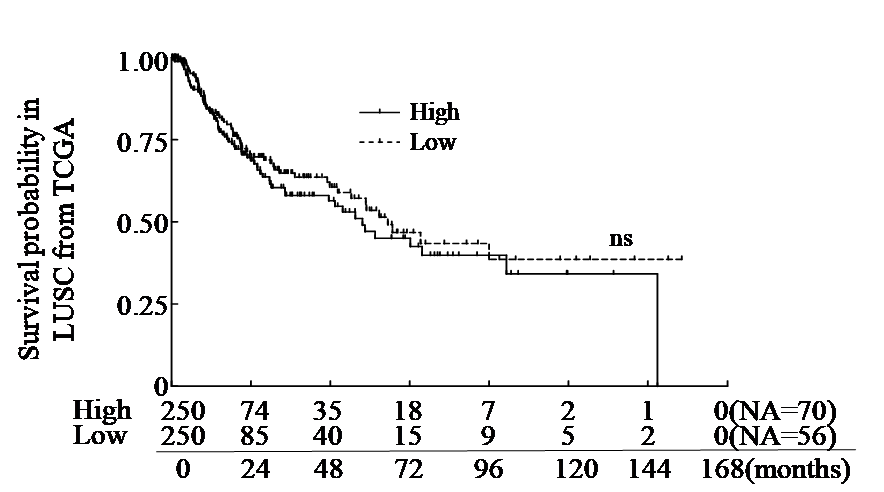


**Fig. S5** Alkaline phosphatase (ALPL) expression in LUSC patients in the TCGA database is not related to prognosis. The relationship between ALPL expression in LUSC patients in the TCGA database and the disease-free survival rate. ns, no statistical significance (*P*> 0.05).


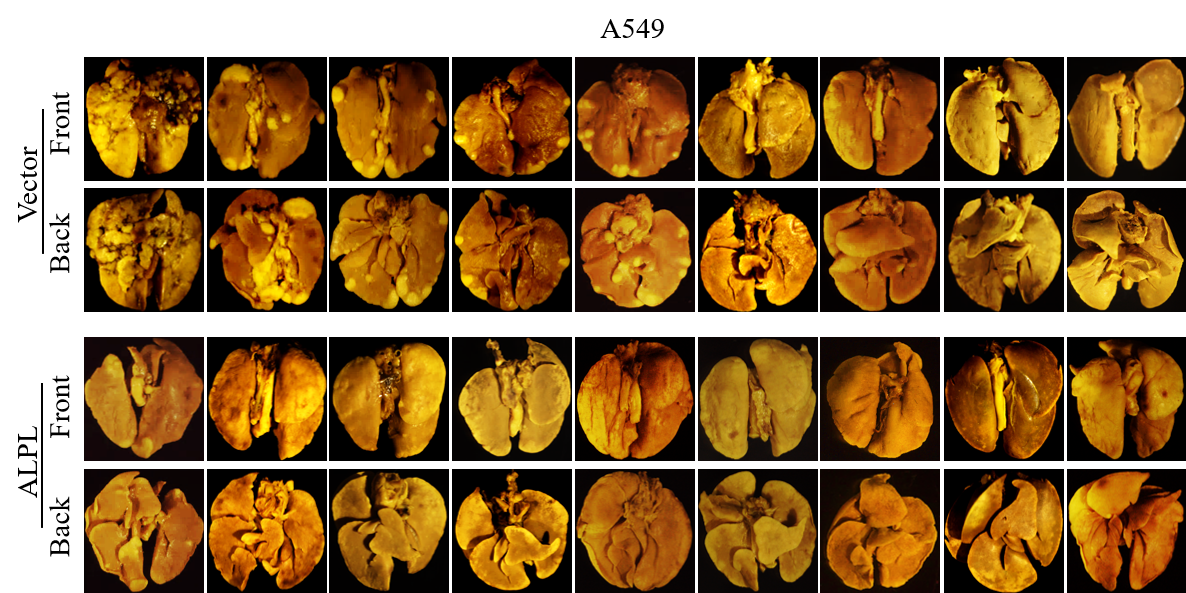


**Fig. S6** Alkaline phosphatase (ALPL) inhibits the metastasis of A549 cells *in vivo*. A tail vein injection model was used to evaluate lung metastasis. The front and back images of the lungs are shown, n=9.

**
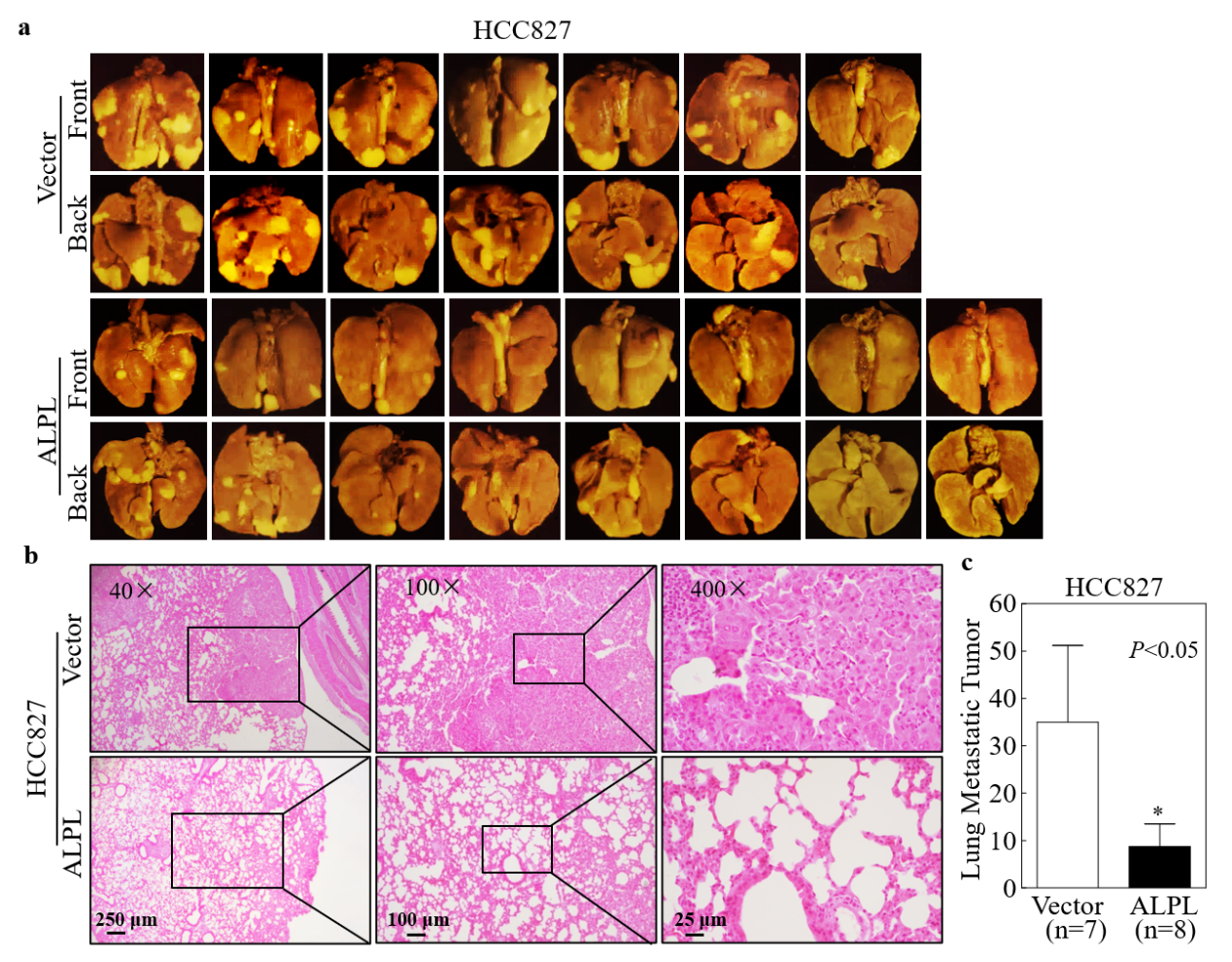
**

**Fig. S7** Alkaline phosphatase (ALPL) inhibits the metastasis of HCC827 cells *in vivo*. (**a**) A tail vein injection model was used to evaluate lung metastasis. The front and back images of the lungs are shown, HCC827(Vector) n=7, HCC827(ALPL) n=8. (**b**) Representative H&E images of HCC827 (vector) and HCC827 (ALPL) tumors. (**c**) Data represent the numbers of metastatic lung tumors of nude mice as indicated. Asterisks (*) represent statistical signiﬁcance (*P*< 0.05).


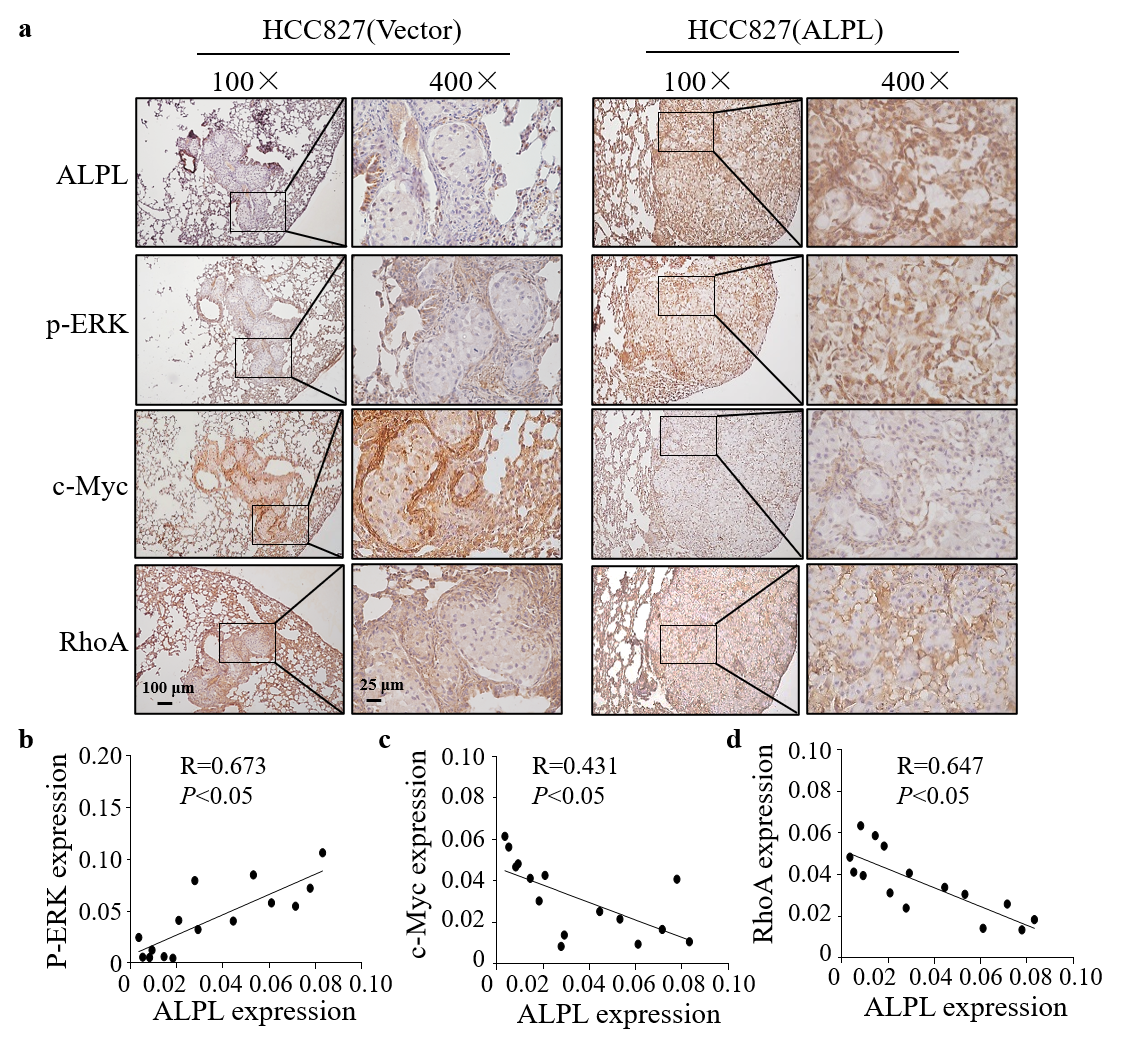


**Fig. S8** Alkaline phosphatase (ALPL) inhibits the metastasis of LUSC cells *in vivo*. (**a**) A tail vein injection model was used to evaluate lung metastasis. The front and back images of the lungs are shown.
